# Platelets and Metastasis: New Implications of an Old Interplay

**DOI:** 10.3389/fonc.2020.01350

**Published:** 2020-09-18

**Authors:** Serena Lucotti, Ruth J. Muschel

**Affiliations:** ^1^Children's Cancer and Blood Foundation Laboratories, Departments of Pediatrics, and Cell and Developmental Biology, Drukier Institute for Children's Health, Meyer Cancer Center, Weill Cornell Medicine, New York, NY, United States; ^2^Cancer Research UK and MRC Oxford Institute for Radiation Oncology, Department of Oncology, University of Oxford, Oxford, United Kingdom

**Keywords:** platelets, megakaryocytes, cancer metastasis, coagulation, anti-coagulant therapy, thromboembolism

## Abstract

During the process of hematogenous metastasis, tumor cells interact with platelets and their precursors megakaryocytes, providing a selection driver for the metastatic phenotype. Cancer cells have evolved a plethora of mechanisms to engage platelet activation and aggregation. Platelet coating of tumor cells in the blood stream promotes the successful completion of multiple steps of the metastatic cascade. Along the same lines, clinical evidence suggests that anti-coagulant therapy might be associated with reduced risk of metastatic disease and better prognosis in cancer patients. Here, we review experimental and clinical literature concerning the contribution of platelets and megakaryocytes to cancer metastasis and provide insights into the clinical relevance of anti-coagulant therapy in cancer treatment.

## Introduction

Besides their role in hemostasis and wound healing, platelets are a key player during metastatic spread. The idea that platelets support tumor cells during their metastatic endeavors is not new. In 1865, the French doctor Armand Trousseau observed excessive blood clotting in patients with occult carcinoma, including himself, and defined it as the Trousseau syndrome (1, 2). One century later, Gasic showed for the first time that platelets are a prerequisite for experimental metastasis (3, 4). Since then, numerous studies have confirmed the relationship between thrombosis and metastasis. Experimental depletion of platelets by pharmacological or genetic means almost completely abrogates metastasis in a number of mouse models, including but not limited to (3, 5–9). Moreover, inhibition of platelet aggregation through different means, including vitamin K agonists, thrombin inhibitors, non-steroidal anti-inflammatory drugs (NSAIDs), and genetic platelet activation deficiency decrease metastatic colonization to the lungs in experimental models (4, 8–15), supporting the notion that platelet aggregation is a pivotal event during metastatic seeding. In the clinical setting, thrombocytosis (high platelet count, >450,000/mL) and platelet hyperreactivity are associated with cancer progression and a worse prognosis. This correlation has been confirmed across different cancer types, including pancreatic, colorectal, lung, and breast cancer (16–21). Compelling evidence also comes from clinical trials showing reduced risk of metastatic disease in patients under anti-coagulant therapy (22). Nevertheless, anti-coagulant therapy is not yet a component of standard cancer treatment or prophylaxis.

So, what is the role of platelets during the metastatic cascade? Decades of research have uncovered a multifaceted interplay between platelets and tumor cells, whereby platelets directly interact with tumor cells in the bloodstream and support many different aspects of their dissemination. This review first summarizes the biology of platelets and their precursors megakaryocytes and then examines the interaction of tumor cells with platelets and megakaryocytes during the metastatic cascade, including its role in the onset of thromboembolic disease in cancer. Finally, we provide a perspective on the current status of adjuvant anti-coagulant therapy in cancer treatment, highlighting its limitations, and future potential.

## Atypical Myeloid Cells: Platelets and Megakaryocytes

Platelets are small membrane bound, blood-borne cell fragments of 2–3 μm in diameter, with a distinctive discoidal shape and lacking a nucleus. They are one of the most abundant type of cells in the blood circulation (150–350 × 10^6^/mL in humans and 1,100 × 10^6^/mL in mice) (23, 24). Thanks to their small size and shape, platelets preferentially marginate toward the outer edge of the blood stream, where lower shear rate and the proximity to vascular endothelium maximizes platelet responsiveness to vascular damage (25–27). Platelets contain numerous cytoplasmatic secretory granules: α-granules and dense granules. The peripheral membrane of α-granules (~50–80 per platelet) has receptors and proteins that are involved in platelet adhesion, angiogenesis and recruitment of immune cells. Dense granules (~3–8 per platelet) contain the low molecular weight agonists of platelet aggregation, involved in the activation, and recruitment of additional platelets to the site of damage (24, 28).

Being anucleate, platelets cannot generate new mRNA and have a short half-life of 8–10 days in humans and 3–5 days in mice. In order to maintain an adequate platelet count, approximately 100 billion platelets are produced every day from their myeloid precursors megakaryocytes (29). Megakaryocytes are giant (50–100 μm in diameter) and very rare cells (~0.01% of bone marrow nucleated cells) that differentiate from common myeloid progenitor cells within specialized osteoblastic and perivascular niches of the bone marrow (30, 31). During development and under pathological conditions megakaryocytes may also be found in the liver and spleen (32–34). Recently, Lefrançais et al. have shown that megakaryocytes of extrapulmonary origin also reside within the lung vasculature in mice, providing evidence that lungs are an active site of platelet biogenesis (35).

### Megakaryocytes and Thrombopoiesis

The production of platelets is initiated by megakaryocytes through a multistep maturation and developmental process. In response to thrombopoietin (TPO), the amount of cytoskeletal proteins, intracellular granules, and membrane lipids increases in megakaryocytes, leading to a massive enlargement of cytoplasmatic volume accompanied by the formation of an invaginated membrane system (IMS). Concomitantly, numerous DNA replication cycles occur in the absence of cell division (endomitosis), generating a polyploid mature megakaryocyte (35, 36). It is believed that endomitosis serves to increase the amount of lipids, mRNA, and proteins to be transferred to the resulting platelets. Finally, mature megakaryocytes produce membrane and cytoplasmatic protrusion into the blood vessels called preplatelet and proplatelets (35, 36). This process is highly dependent on the reassembly of cytoskeletal filaments that occurs during megakaryocyte maturation and which controls proplatelet elongation and the transportation of granules and organelles from the megakaryocyte cytoplasm into the proplatelet tips. Mature platelets are then produced by spontaneous rounds of fragmentation of proplatelets in the circulation (30, 36). In the bone marrow, thrombopoiesis takes place in the vascular niche and requires the interaction of megakaryocytes with bone marrow endothelial cells and the perivascular extracellular matrix (ECM), as reviewed by (37).

### Platelets and Hemostasis

The main functions of platelets are to maintain the integrity of the vascular system by arresting bleeding (hemostasis) and promoting wound healing at sites of vascular injury. Hemostasis involves two parallel and interrelated processes at the site of damage: thrombosis, which is the formation of a platelet aggregate (thrombus), and coagulation, a cascade of cell activation and proteolytic reactions involving different cell types (endothelial cells, platelets, and leukocytes) and soluble proteins (coagulation factors). Coagulation factors are enzymes with serine protease activity present in the circulation as inactive zymogens that undergo activation through proteolytic cleavage (38). These processes culminate with the generation of thrombin, which cleaves soluble fibrinogen into fibrin and leads to the deposition of fibrin fibers and the formation of a (fibrin) clot, a plug of platelets and fibrin mesh (29, 39).

Upon vascular damage or endothelial retraction, components of the endothelial basement membrane and other sub-endothelial extracellular proteins such as von Willebrand Factor (vWF), collagens and fibronectin are exposed to the blood stream. Interaction of integrin receptors expressed on resting platelets with these exposed ligands induces a rapid cascade that leads to the activation of platelets (within seconds) and the formation of a fibrin clot (within minutes). First, the interaction of vWF/collagen with the multimeric complex glycoprotein (GP) Ib-IX-V on the platelet surface causes platelet tethering and rolling on the exposed subendothelium (40–42). This initial and reversible platelet adhesion is followed by intracellular signaling leading to platelet activation, a complex cellular process associated with the activation of Src kinases, increase of cytosolic Ca^2+^ concentration and the activation of protein kinase C (PKC) and phosphatidylinositol 3 kinase (PI3K) (43, 44). Class I kinases such as PI3Kβ have a pivotal role in Akt phosphorylation and Ca^2+^ mobilization during thrombus formation (45). Ultimately, these cellular responses lead to the rearrangement of the platelet cytoskeleton, culminating in granule secretion, change of cell shape with production of pseudopodia and platelet spreading, and the “inside-out” conformational change of membrane integrin α_2_ß_1_ and α_IIb_ß_3_ (or GPIIa/IIIb) from a low to a high affinity form (24, 29). The high affinity form of these integrins mediate firm adhesion to collagen (α_2_ß_1_), vWF, and fibrin(ogen) (α_IIb_ß_3_), supporting clot stabilization (46, 47). Thrombus amplification is further sustained by the release of adhesion molecules (e.g., vWF and fibrinogen), coagulation factors (e.g., factors V and IX) and soluble agonists (e.g., ADP, serotonin) and the exposure of additional platelet receptors (GPIb-IX-V, α_IIb_ß_3_, GPVI, and P-selectin or CD62P) contained in platelet α- and dense granules (46). Activation of platelets also initiates fatty acid oxidation and the *de novo* synthesis of thromboxane A_2_ (TXA_2_), a secondary mediator of thrombus amplification. Together, ADP, thrombin and TXA_2_ interact with their cognate receptors on other platelets (ADP:P2Y_12_, thrombin:PAR1/4, TXA_2_:TP), leading to platelet activation (24, 39, 48). Thus, the initial layer of activated platelets serves as a reactive surface for the tethering, activation, and aggregation of additional platelets.

Other cell types take part in the generation of a fibrin clot, by either serving as an adhesion surface for platelet tethering or by engaging in the coagulation cascade. Activated endothelial cells at the site of vascular injury locally synthesize or expose endothelial cell leukocyte adhesion molecules. Exocytosis of Weibel-Palade bodies in endothelial cells releases vWF, which in turn binds to platelet GP Ib-IX-V and integrin α_IIb_ß_3_, and exposes P-selectin, which binds to platelet GPIbα and P-selectin ligand (PSGL-1) (42, 49), and E-selectin, which recruit myeloid cells to the site of injury (50, 51). Recruited monocytes, activated endothelial cells and other sub-endothelial stroma cells, such as smooth muscle cells and fibroblasts, express tissue factor (TF, also known as CD142 or thromboplastin), which is the main activator of the coagulation cascade *in vivo*. When exposed to the blood circulation, TF activates coagulation factor VII and binds to its active form VIIa in a bimolecular complex (TF-VIIa) that initiates the coagulation cascade leading to the activation of thrombin and fibrin deposition (52). Thrombin also cleaves protease activated receptor (PAR)1 and PAR4 on platelets, inducing their activation and aggregation (48). Ultimately, to complete healing, the fibrin clot will be dissolved by fibrinolysis [as reviewed by (53)].

Although platelet have been traditionally studied in the context of blood coagulation, we now know that they are also involved in many other processes such as inflammation, angiogenesis, and innate immunity. These functions have been reviewed by others (54–56) and will not be the focus of the current review.

## Tumor Cell-Platelet Interactions

**Figure 1:**
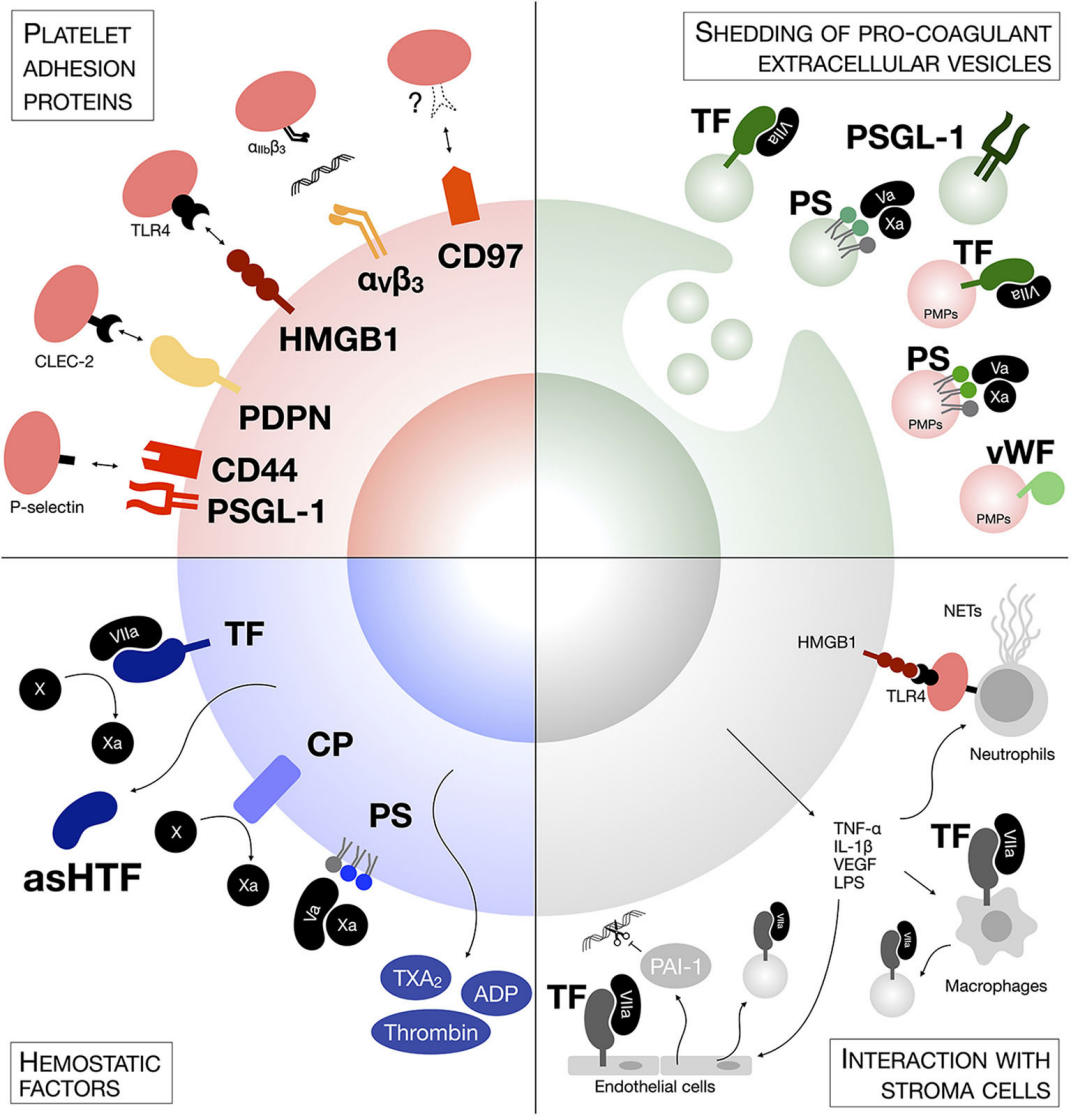
Mechanisms of tumor cells-platelets interaction. Tumor cells generate platelet activation and the formation of microclots on their surface through several mechanisms, including expression of hemostatic factors and adhesion proteins, either on their surface or on the surface of shedded extracellular vesicles, or though the generation of a pro-thrombotic intravascular metastatic niche involving other stroma cells.

Tumor cells have adapted to mimic some steps of the hemostatic process and they interact with circulating platelets during their hematogenous transit (Figure 1). This interaction happens within minutes from tumor cell intravasation (7, 57) and relies on an expression pattern triggered by classical oncogenic mutations and microenvironmental cues [as reviewed by (58)].

### Hemostatic Factors

Different cancer cells express a variety of hemostatic factors. TF has been found constitutively expressed by most tumor cell lines and metastatic cells express up to 1,000-fold higher levels of TF in comparison to non-metastatic cells (59–61). Epithelial-to-mesenchymal transition (EMT) can also induce TF expression in circulating tumor cells (CTCs) (62). The levels of TF expression on tumor cells correlate with cancer progression and poor prognosis (63). TF-interacting coagulation factor VII has also been found overexpressed in colorectal cancer and hepatocellular carcinoma cells and correlated with hepatic metastasis (64, 65). In addition to engaging the coagulation cascade, both TF and factor VII induce intracellular signaling that supports tumor cell growth, invasion and migration (52, 64, 66). Along the same lines, cancer procoagulant (CP) is a cysteine protease that directly cleaves and activates coagulation factor X, thus inducing the exogenous coagulation cascade and ultimately platelet activation (67, 68). CP is expressed in human malignant tissues, but largely absent in normal tissues (69–71).

Proteins are not the only hemostatic factors in tumor cells; the asymmetric distribution of membrane phospholipids on cancer cells is also responsible for coagulation. The membrane phospholipid phosphatidylserine (PS), which is preferentially localized in the inner leaflets of normal eukaryotic cell membranes (72), has been found highly exposed on the outer leaflet of tumor cells and this exposure is linked to mutations in phospholipid translocases such as flippase (73–75). Intriguingly, metastatic cells have lower flippase activity and thus higher level of PS than their non-invasive counterparts (75). Once on the outer membrane leaflet, the anionic PS creates a negatively charged surface that binds factors Va and Xa, thus initiating the assembly of the prothrombinase complex, and supports the conformational change that activates their proteolytic activity, leading to thrombin deposition, and platelet aggregation (76).

Hemostatic factors are not only expressed on the surface of tumor cells, but they are also released in their soluble form to create a pro-thrombotic niche. Human tumor cell lines secrete a soluble alternatively spliced form of TF (known as asHTF) (77, 78), which can also be found in the plasma of cancer patients (79). Additionally, platelet agonists such as ADP, TXA_2_, and thrombin have been detected in cell line supernatants and cancer biopsies (80–82) and interact with platelet receptors P2Y_12_, TP, and PARs, respectively, to initiate platelet aggregation. Hence, tumor cells can activate platelet aggregation also through a paracrine route.

### Platelet Adhesion Proteins

Tumor cells express binding proteins that mediate direct activation and adhesion of platelets in the absence of other plasma components. PSGL-1 on tumor cells directly interact with P-selectin exposed on activated platelets (11, 83). Other glycoproteins bearing sialyl-Lewis^x^ structures on the tumor cell surface have also been shown to mediate adhesion to P-selectin-expressing platelets (11). Tumor cell CD44 is also involved in P-selectin binding, either directly or via fibrin (84). Although P-selectin-PSGL-1 engagement requires prior activation of platelets, elegant studies by Furie and Furie using P-selectin- or PSGL-1-null mice suggest their role in fibrin generation together with TF deposition in the growing thrombus (85, 86). Thus, the direct association of tumor cells with platelets through a P-selectin-PSGL-1 interaction is sufficient to mediate the deposition of a fibrin clot on the surface of tumor cells. Podoplanin (PDPN, tumor cells): CLEC-2 (platelet) and HMGB1 (tumor cells): TLR4 (platelets) are other adhesion protein-ligand pairs that support platelet activation and aggregation on tumor cells, and ultimately metastasis (87–91). Additionally, integrin α_IIb_β_3_ on activated platelets has a central role in adhesion to melanoma and breast cancer cells by interacting with tumor cell integrin α_V_β_3_ via fibrinogen (92–94). It is interesting to notice that α_V_β_3_ expression confers a proliferative advantage to breast cancer tumor cells during early stages of brain metastasis, suggesting that platelet promote the survival of cells with higher metastatic potential during their hematogenous transit (95, 96). More recently, Ward et al. have identified the G protein-coupled receptor (GPCR) CD97 on tumor cells as a novel binding protein mediating interaction with platelets. CD97 induces α_IIb_ß_3_-dependent platelet activation and their aggregation on the surface of tumor cells, although its cognate receptor on platelets still remains to be described (97).

### Shedding of Pro-coagulant Extracellular Vesicles

Both hemostatic factors and platelet ligands can be shed by tumor cells in extracellular vesicles, in particular microparticles (MPs, also called microvesicles). MPs are small membrane vesicles of 100–1,000 μm in diameter released by direct membrane budding (98). Already in 1981, Dvorak et al. detected the presence of tumor-derived MPs *in vitro* and in ascitic fluids of tumor-bearing animals. These MPs could induce fibrin deposition *in vivo* (99). Tumor cells have been found responsible for the production of these pro-coagulant MPs. MPs expressing TF, PSGL-1, and PS can be detected in the culture medium of tumor cells and in tumor-bearing mice, and mediate thrombin generation and thrombus growth *ex vivo* and *in vivo* (77, 100–103). These MPs accumulate in the growing thrombi through a PSGL-1-mediated mechanism and accelerate thrombus growth (86, 100). MPs are found in the blood of cancer patients. Patients with pancreatic, colorectal, brain, prostate, and breast cancer have higher levels of plasma TF/PS-expressing MPs and higher MP-associated pro-thrombotic activity than healthy subjects, especially during advanced stages of disease and after chemotherapy or radiotherapy (104–110). Metastatic cancer patients had particularly high plasma levels of TF^+^ MPs across a range of cancer types (109).

Certainly not all pro-coagulant MPs in the blood of cancer patients derive from tumor cells. Platelet-derived MPs (PMPs) are the most represented population of MPs in plasma from healthy individuals, accounting for up to 90% of circulating MPs (111–113). Although resting platelets can release MPs (114), most PMPs are produced as a result of platelet activation (111, 115), and are involved in thrombus expansion during hemostasis through the expression of PS, TF, and vWF on their surface (112, 116–118). PMPs are elevated in murine models of cancer and in cancer patients (100, 109). Hence, it is possible that by stimulating platelet aggregation, CTCs induce an increase in platelet-derived MPs, contributing to the pool of circulating pro-coagulant MPs. Interestingly, TF-expressing MPs are also detectable in healthy people, but are not associated with apparent pro-coagulant activity (39). MPs derived from resting platelets lack P-selectin expression, a marker of platelet activation and pro-coagulant protein (111). Hence, cancer-derived MPs and PMPs might express TF in an alternative and readily active conformation, or TF association with negatively charged PS or other adhesion proteins might be required to exert its pro-coagulant function.

Exosomes are a different type of extracellular vesicles of 30–150 nm in diameter that originate in the endocytic pathway and have a pivotal role in mediating short- and long-distance intercellular signaling in both physiological and pathological conditions (119). Although previous evidence suggests that cancer-derived exosomes may initiate thrombosis *in vitro* and *in vivo* (120–122), the mechanism and prognostic value of these extracellular vesicles still remains unknown.

### Interaction With Stroma Cells

As well as directly activating platelets, cancer cells promote a procoagulant niche by altering the thrombotic phenotype in other neighboring stroma cells. Pro-inflammatory cytokines [i.e., TNF-α and interleukin (IL)-1β] and pro-angiogenic factors (i.e., VEGF) released by tumor cells induce the overexpression of TF by endothelial cells and monocytes (123–128) and the release of vWF by endothelial cells (129). Moreover, tumor cell IL-1 induces endothelial secretion of plasminogen activator inhibitor (PAI)-1, an inhibitor of fibrinolysis (130). Tumor cell-derived pro-inflammatory cytokines also induce a peak of stroma-derived MPs contributing to thrombus growth. LPS-stimulated monocytes and endothelial cells release pro-coagulant MPs expressing TF and PSGL-1 (118) and higher number of endothelial-derived MPs can be found in the blood of cancer patients (100, 109). Additionally, tumor cells disseminating to the lung and liver recruit and activate neutrophils to release of extracellular DNA traps (NETs) intravascularly (131, 132). NETs induce platelet aggregation through PS exposure, PMP accumulation and endothelial cell activation, and are associated with increased hypercoagulability and risk of venous thromboembolism in cancer patients (133–135). Interestingly, platelets interacting with tumor cells through the TLR4 axis can also activate NETs formation through a P-selectin-dependent mechanism (136, 137), further amplifying thrombosis.

## Platelets and Metastatic (In)efficiency

**Figure 2:**
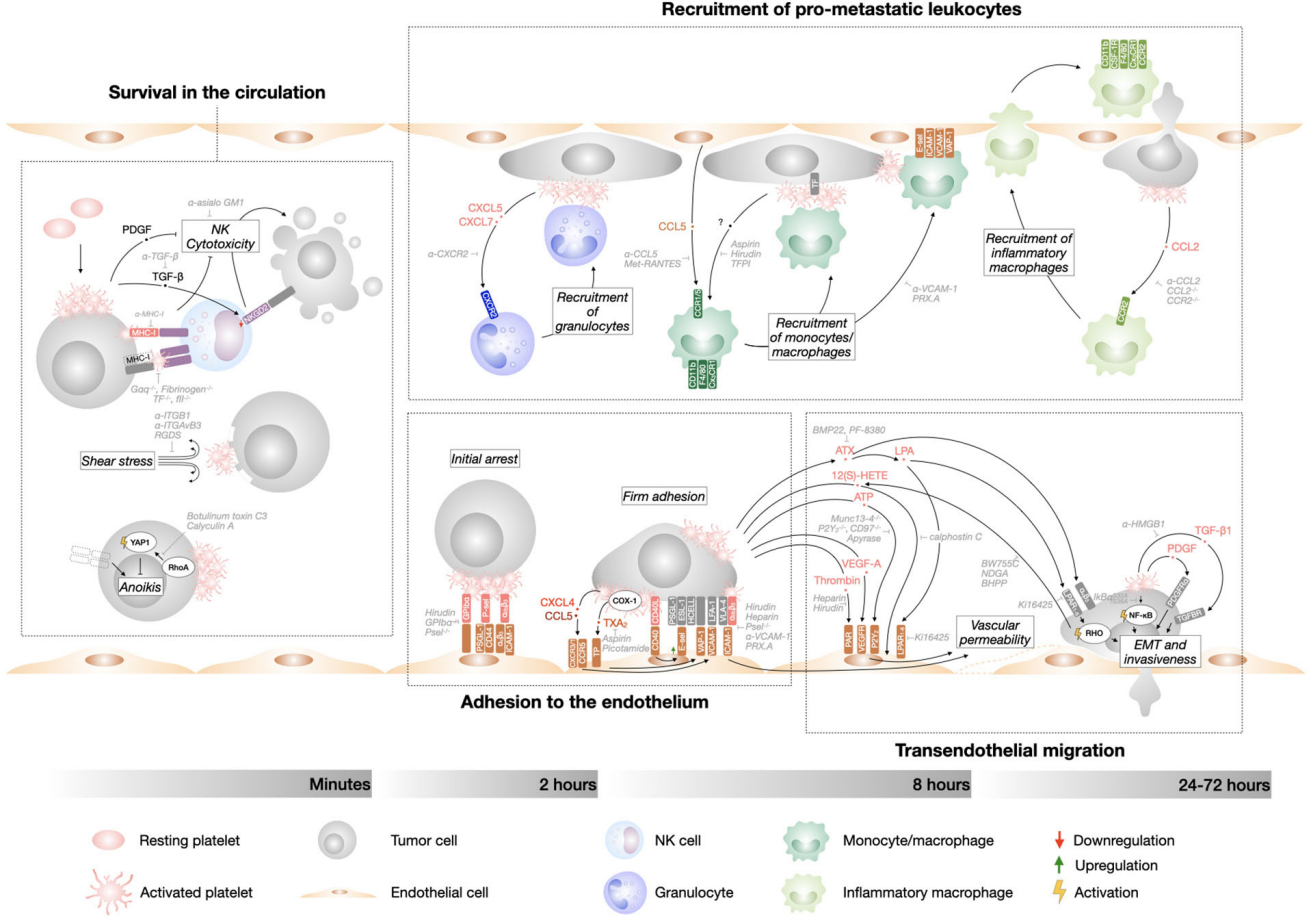
Tumor cell-platelet interplay during the metastatic cascade. Diagram depicting the intravascular steps of the metastatic cascade that are supported by platelet interaction with tumor cells and their timeline. Therapeutic approaches that interfere with the different steps of tumor cells dissemination are indicated in gray.

*Per se*, metastasis is a highly inefficient process. Experimental evidence suggests that <0.02% of tumor cells entering the circulation end up forming a macroscopic metastases, either in the lungs (0.01% of cells) (138) or liver (0.018% of cells) (139). There are different bottleneck events during the metastatic cascade that reduce the ability of tumor cells to colonize a distant organ. Seminal work by Fidler showed that ^125^IUDR-labeled B16 melanoma cells arrest in the lungs shortly after intravenous introduction, but only 1% of the injected tumor cells survived in the lungs during the first 24 hours, with most tumor cells dying during the first hour after injection (138). These findings have been confirmed in other models (140, 141) and identified that metastatic inefficiency happens mainly during the intravascular phase of tumor cell dissemination. The circulatory system is in fact a very hostile environment and CTCs are exposed to cell death through immunological, cellular, and physical means. Despite the strong negative selection against tumor cells during the first hour in the blood stream, the intrinsic inefficiency of the metastatic process is not sufficient to abrogate the appearance of distant metastasis. It has been estimated that millions of tumor cells detach from the primary tumor and enter the circulation every day. On such a large scale, 0.02% of surviving cells is no longer a small number, explaining why metastasis is far from rare in cancer patients. A large body of evidence clearly point to platelets-tumor cell interaction as the main reason for tumor cell survival during the intravascular phase of metastasis. Already in 1984, Gorelik et al. had shown that the anti-coagulant heparin dramatically increased the rate of tumor cell elimination during the first day after their injection (142). Platelet depletion during tumor cell presence in the circulation drastically impaired metastatic burden (7, 143). More recently, we have shown that anti-platelet therapy during the intravascular phase of metastasis, but not after tumor cell extravasation, was sufficient to reduce the number of pulmonary metastatic foci subsequently formed (9). Over the years, many mechanisms have been documented for the supporting role of platelets during metastasis, involving a crosstalk between tumor cells and platelets directly or through other stroma cells (Figure 2). Platelets have been found to assist multiple consecutive steps of the metastatic cascade, including tumor cell survival, interaction with the endothelial and immune cells, and transendothelial migration. It is widely accepted that both physical tumor cell-platelet interactions and activation of intracellular signaling pathways in both cell types support these steps of metastasis.

### Tumor Cell Survival in the Circulation

Patrolling natural killer (NK) cells are the main form of anti-tumor immunosurveillance in the metastatic cascade. NK depletion dramatically increases metastasis to lungs and liver (15, 143–145). Loss of class I major histocompatibility complexes (MHC-I, “missing self”) and up-regulation of surface proteins (“altered-self”) on tumor cells are recognized by NK cells and elicit an anti-tumor response. Cell killing takes place through different mechanisms, including release of cytotoxic granules, engagement of death receptors and secretion of tumor suppressant IFN-γ (146). Platelet cloaking of tumor cells can prevent NK-dependent tumor cell cytolysis (12, 13, 142–144, 147). This concept was firstly elaborated by Nieswandt et al. who showed that the reduction of lung metastasis induced by thrombocytopenia is abrogated by NK cell depletion (143). Interestingly, after tail vein injection of tumor cells, NK depletion supports tumor cell colonization of both lungs, the first vascular bed that they encounter, and liver (142, 143), suggesting that liver colonization in these experimental metastasis models is mainly prevented by NK cell lytic activity. Different mechanisms have been described that explain the NK-suppressive effect of platelets. Platelet express high levels of MHC-I and by coating tumor cells they provide an MHC-I “pseudoexpression” that rescues tumor cell “missing self” and protects them from NK cells recognition (148). TF-dependent platelet and fibrinogen coating have also been found responsible for NK cell evasion (12), potentially via physical shielding of “altered self” and “missing self” expression from immune recognition. Furthermore, activated platelets release soluble factors that induce NK cells quiescence in a paracrine manner. Platelet-derived growth factor (PDGF) inhibit NK cells cytotoxic activity (149, 150) and platelet-derived transforming growth factor (TGF)-ß induces the downregulation of natural killer group 2, member D (NKG2D), a NK cell immunoreceptor that activates anti-tumor reactivity (151).

Additional to NK cells cytotoxicity, hemodynamic shear forces experienced by CTCs in the blood stream induce mechanical damage and death of tumor cells, mainly through increased sensitivity to TNF-related apoptosis-inducing ligand (TRAIL) on NK cells and Smad-dependent cell cycle arrest (152–154). Further, CTCs can undergo cell death due to anoikis, a particular type of apoptosis induced by the disengagement of cell-cell or cell-ECM contacts (155). EMT of CTCs and their clustering in circulating tumor microemboli might provide anoikis resistance (156). The decoration of tumor cells by platelets attenuates tumor cell membrane damage due to shear stress (157). Moreover, platelets bound to tumor cells provide resistance to anoikis by supplying cell-cell contacts and by inducing RhoA-dependent YAP1 activation, which further promotes metastasis (8).

### Adhesion to the Endothelium

Even when tumor cells survive their first hours in the blood stream, their ability to metastasize is entirely reliant on forming stable interactions with the vascular wall, followed by extravasation. Most tumor cells arrest in capillaries, where they can be trapped due to size restriction, but they can also be found adhered to pre-capillary arteries and portal venules, which are larger in diameter, suggesting the existence of active receptor-ligand interactions (158). The initial and transient interaction of tumor cells with the vessel wall can be mediated by the expression of the selectin family of adhesion molecules, including endothelial P-selectin and E-selectin, which establish and disengage low-affinity bonds that make leukocytes and tumor cells appear to “roll” on the endothelium, at least *in vitro* (159). Subsequently, firmer adhesion between tumor cells and endothelium is achieved through the expression of a second subset of adhesion molecules, mainly (but not exclusively) integrins and their ligands (159, 160). The principal endothelial integrin ligands are vascular cell adhesion molecule 1 (VCAM-1) and intercellular adhesion molecule 1 (ICAM-1). vWF is also secreted via exocytosis of endothelial cell intracellular granules, the Weibel-Palade bodies (161). Further, basement membrane exposed after endothelial retraction might be another site of anchorage (162). Under physiological conditions, the expression of selectins and integrin ligands on endothelial cells (a process known as endothelial activation) is tightly controlled by transcriptional regulation, exocytosis of intracellular granules and proteolytic cleavage to avoid inappropriate thrombosis or leukocyte recruitment (159).

A growing body of literature describes the role of platelets in supporting the interaction of tumor cells with the vessel wall. Im et al. have shown that tumor cells were less likely to flatten/spread on lung endothelial cells if mice were anticoagulated with hirudin (57). Platelets express adhesion proteins that mediate their rolling (resting platelets) and adhesion (activated platelets) to the vascular wall (163), and thus may form “sticky” bridges between tumor cells and activated endothelial cells or endothelial basement membrane components. For example, Jain et al. have shown that platelets lacking GPIbα, a member of the vWF-binding GPIb-IX-V complex, can still interact with B16 melanoma cells, but dramatically hinder B16 experimental metastasis. This pro-metastatic activity relies solely on the extracellular domain of GPIbα, suggesting that platelet coating of tumor cells and endothelium adhesion, independent of platelet activation, can support metastasis (164). P-selectin on activated platelets interacting with tumor cells mediate their adhesion to endothelial cells and P-selectin^−/−^ mice or platelets support significantly less lung metastasis (83, 145, 165, 166), although infusion of P-selectin^+/+^ platelets after tumor cell injection partially recovers metastatic nodules, suggesting that platelet P-selectin acts as a pro-metastatic adhesion molecule to both endothelial and tumor cells (167). Integrin α_IIb_ß_3_, which is found on tumor cells-activated platelet clusters, mediates binding to endothelial cells via ICAM-1 and α_V_β_3_ (163, 165, 168).

The rolling and adhesion of platelet-tumor cell emboli requires activation of endothelial cells, as shown by evidence that genetic depletion or pharmacological inhibition of P-selectin and vascular adhesion protein-1 (VAP-1) expression in endothelial cells dramatically hinders metastasis in different platelet-proficient *in vivo* models (83, 145, 165, 169, 170). Platelet microthrombi on tumor cells induce endothelial cell expression of E-selectin, VCAM-1, and VAP-1 in lung endothelial cells and anti-coagulant therapy dramatically reduces endothelial activation to basal levels (9, 170, 171). Both direct and paracrine signaling have been found responsible for platelet-dependent initiation of endothelial cell activation. Direct interaction of CD40L on activated platelets with endothelial CD40 triggers expression of E-selectin, VCAM-1, and ICAM-1 on endothelial cells *in vitro* and *in vivo* (172). Interestingly, CD40L on T cells mediates their interaction with antigen presenting cells and B-cells, suggesting that activated platelets might also have a role in linking innate and adaptive immune responses. Additional to direct contact, pro-inflammatory cytokines released by activated platelets are known activators of endothelial cells, for example CXCL4 and CCL5 (173). Our lab has shown that TXA_2_ derived from platelets interacting with tumor cells intravascularly induces the expression of E-selectin and VCAM-1 by endothelial cells in the proximity of tumor cells-platelets emboli (9). Tumor cells can then establish firm adhesion with activated endothelial cells via expressed selectin ligands [e.g., PSGL-1, CD44v, CD24, HCELL, E-selectin ligand 1 (ESL-1); (174–178)] and integrins [e.g., α_4_ß_1_/VLA-4, α_L_ß_2_/LFA-1, and α_3_β_1_; (162, 179–182)].

It is important to mention that the initial arrest of tumor cells, in particular in pulmonary circulation, seems to be largely independent of platelet interaction with tumor cells. Although the association of tumor cells α_V_β_3_ with platelets α_IIb_β_3_ mediate their adhesion to collagen I *in vitro* and increase lung metastasis *in vivo* (94, 183), its role in the adhesion to endothelial cells is not understood. Heparin treatment (142) or platelet depletion (7) does not affect the number of tumor cells arrested in the lungs at 10 minutes after injection, suggesting that the physical entrapment of tumor cells in the lung capillaries remains a main mechanism of initial arrest.

### Recruitment of Pro-metastatic Leukocytes

The interaction of tumor cells with leukocytes, including myeloid cells and lymphocytes, has been observed for a long time. More recently, evidence has emerged indicating that platelets play a pivotal role in coordinating the formation of transient tumor cell-immune cell intravascular niches. Labelle et al. have shown that early after intravasation, tumor cells lodging in the lung vasculature are found decorated with granulocytes (CD11b^+^ MMP9^+^ Ly6G^+^) that support metastatic seeding and subsequent metastasis (7). Importantly, tumor cell interaction with platelets leading to release of soluble chemoattractants is necessary for the recruitment of these granulocytes, including the granulocyte CXCR2 ligands CXCL5/7. Neutrophils might be part of these early microemboli, as they are recruited by platelet-derived chemoattractants and adhere to activated platelets and endothelium (184). Platelet-neutrophil interaction through TLR4 and P-selectin and platelet-derived TXA_2_, CXCL4, and vWF induce intravascular NETosis, which greatly supports seeding and progression of pulmonary metastasis (132). Tumor cells actively adhere to intravascular NETs through β1 integrin in hepatic sinusoids (185), suggesting that NETs-dependent trapping could support tumor cell arrest in much larger vessels than capillaries. Tumor cell-platelet-granulocyte emboli start to dissolve after 4 hours and are followed by a second wave of immune cells recruitment driven by platelets. Gil-Bernabe et al. have described the recruitment of a subset of undifferentiated monocytes/macrophages (CD11b^+^ F4/80^+^ CX_3_CR1^+^ CD11c^−^ Ly6C^−^) to disseminating tumor cells (15). This recruitment depends on TF expression by tumor cells, leading to the deposition of microclots on tumor cells that establish direct interaction with the monocytes. Microemboli containing monocytes/macrophages, tumor cells, and platelets form within the vasculature from 2 hours after tumor cell introduction and reach their maximum volume at 8 hours, but are dissolved by 24 hours. The recruitment of these myeloid cells is promoted by the release of CCL5 by activated endothelial cells and their expression of the adhesion molecules VCAM-1, VAP-1, and E-selectin, all induced by clots on tumor cells (50, 170, 171). Selective depletion or functional impairment of monocytes/macrophages significantly reduced tumor cell number during the first day post-injection, suggesting that patrolling monocytes/macrophages support tumor cell survival in the circulation during early phases of metastasis (15). Finally, a third wave of inflammatory monocytes are recruited to tumor cells through CCL2-CCR2 signaling (186). Platelets are a main source of CCL2, which is stored in their α-granules (96). After diapedesis, these monocytes differentiate into metastasis-associated macrophages (MAMs), characterized by an inflammatory phenotype (F4/80^+^ CSF1-R^+^ CD11b^+^ Ly6C^−^ CX3CR1^high^ CCR2^high^) (187). These MAMs are localized in the extravascular space and are recruited to the proximity of extravasating tumor cells within 24–72 hours after injection, and support tumor cell extravasation and initial growth (186, 187). Although tumor cell-platelet interaction still takes place at the moment of initial MAM recruitment, it is still unknown if platelets are involved in their chemotaxis and differentiation.

Interestingly, the recruitment of pro-metastatic immune cells by platelets in metastatic organs can happen before tumor cell colonization, at the level of pre-metastatic niche. Our lab has shown that the population of Cx_3_CR1^+^ monocytes/macrophages in the lungs increases in pre-metastatic tumor bearing mice, but not in mice injected with TF-deficient tumor cells or in mice that are anticoagulated with aspirin or hirudin during lung preconditioning (9, 15). Hence, circulating platelets activated at the primary tumor can carry signals to distant organs, supporting tumor cell homing.

### Transendothelial Migration (TEM)

Once adhered to the vascular wall, tumor cells cross the endothelial barrier in a process called transendothelial migration (TEM), which happens during the first 3 days after entering the circulation, although the timing is highly dependent on the cell type and the secondary organ (188). Similar to leukocyte diapedesis, tumor cell TEM preferentially takes place by a paracellular route and the acquisition of an invasive phenotype and is accompanied by an increase in vascular permeability (159). Several mechanisms have been proposed to account for the increase in vascular permeability during metastatic seeding, including transient disruption of endothelial cell junctions (189), cytoskeletal rearrangements leading to endothelial retraction (159), and apoptosis/necroptosis of endothelial cells, resulting in the irreversible opening of the endothelial barrier (190, 191). Vascular permeability is induced locally in the proximity of tumor cell-platelet microemboli and might be potentiated by further exposure of subendothelial vWF, TF, and collagen. Thrombin interaction with PAR receptors on endothelial cells induces endothelial cell retraction (192). Adenine triphosphate (ATP) is released by platelet dense granules in response to interaction with tumor cells and can bind to P2Y_2_ receptors on endothelial cells, resulting in the opening of vascular junctions and TEM (97, 193). Similarly, autotaxin (ATX) derived from tumor cell-stimulated platelets and its product lysophosphatidic acid (LPA) interact with tumor cells α_V_β_3_ and LPA receptors (LPAR), respectively, and promote TEM and bone metastasis of breast cancer (194). LPA also increases the permeability of cerebral microvascular endothelial cells directly (195). Furthermore, 12(S)-HETE, a product of arachidonic acid metabolism derived from tumor cells and interacting platelets, induces cytoskeletal rearrangement in endothelial cells, leading to their retraction (196–198). VEGF-A from activated platelets might also induce endothelial permeability and TEM, as does VEGF-A delivered by inflammatory MAMs in lungs (186). Direct contact between tumor cell-platelets clusters and endothelial cells also induces endothelial permeability. Interaction of endothelial VCAM-1 with tumor cell VLA-4 and/or endothelial ICAM-1 with platelet α_IIb_ß_3_ triggers an “outside-in” signaling cascade in endothelial cells that induces the digestion of tight junctions, cytoskeletal rearrangement, and endothelial retraction (199, 200). Caspase-dependent apoptosis of endothelial cells has also been observed in response to bacteria-activated platelets (201). Although endothelial necroptosis has been characterized as a novel mechanism of TEM during metastasis, platelet do not seem to contribute (191).

Concomitant with inducing vascular permeability, tumor cells need to undergo dynamic changes of cell shape to move through the vascular wall. Although it is not a prerequisite for intravasation, EMT of CTCs is associated with the acquisition of mesenchymal markers, allowing greater motility (202), as does the subsequent formation of proteolytically active protrusions called invadopodia through RHO- and ROCK-dependent polymerization of actin fibers at their leading edge (203–205). The acquisition of this invasive phenotype by tumor cells is supported by their association with platelets, as shown by the fact that cancer cells exposed to activated platelets have a higher capacity for ECM degradation and tissue infiltration (206). This phenotype is sustained far longer than the transient interaction between tumor cell-platelet, whose emboli are dissolved within 24 hours (15, 207). Evidence suggests that this process takes place in two-wave kinetics. Initially (within 16 hours), CD97-mediated interaction with platelets induces release of platelet LPA and activates RHO in tumor cells, promoting tumor cell invasiveness (97). Later on (from 40 hours), platelets induce a gene expression signature in tumor cells that initiates EMT and metastatic seeding, including pro-metastatic *mmp9, ccl2*, and *serpine1* (14). Labelle et al. elegantly showed that both the physical interaction with platelets, leading to NF-kB activation in tumor cells, and the paracrine signaling of TGF-β1 released by platelet cooperate to induce this EMT program (14). TGF-β1 is stored in platelet granules (208) and released upon tumor cell dependent platelet activation and direct receptor-ligand interactions, including HMGB1:TLR4 (91). Although the effect of platelet on tumor cell protrusion has not been described, the upregulation of PDGF receptor α (PDGFRα) in tumor cells undergoing EMT has been associated with invadopodia formation and stabilization (205). PDGF is readily released by the α-granules of activated platelets, suggesting that they might initiate invadopodia formation by tumor cells.

### Later Phases of Metastasis: Angiogenesis, Proliferation, and Dormancy

Despite extensive evidence for the facilitation of metastasis by platelets prior to completion of extravasation, the evidence for platelet involvement in later stages of metastasis as a general rule is tenuous. Although platelet depletion decreased the proliferation and viability of tumor cells in mice (209), our lab and others observed that anticoagulant therapy, blockade of platelet activation and of tumor cell-platelet adhesion molecules failed to affect tumor growth both at the primary and at the secondary site (9, 12, 91, 209, 210). Extravascular platelets can be detected in primary tumors, where they support tumor cell proliferation and local invasion (33, 211, 212). It is not known whether they are similarly present in metastases. Although this would seem likely, such evidence has not been confirmed in metastasis models.

A potential role of platelets in angiogenesis has been reported. They support vessel maturation by promoting endothelial junction formation/endothelial VE-cadherin expression (211) and pericyte coverage of vessels (33). Additionally, platelets store or take up anti-angiogenic (e.g., endostatin, thrombospondin-1, and CXCL4) and pro-angiogenic factors (e.g., such as vascular endothelial growth factor (VEGF), epidermal growth factor (EGF), and PDGF), which are segregated in different types of α-granules and selectively released (96, 211, 213–217). The release of pro-angiogenic molecules has been observed both at the primary and at the metastatic site and is associated with better blood perfusion and vessel stability (209, 211). MPs from activated platelets can also support angiogenesis (218). Platelet could potentially affect tumor growth. Growth factors contained in platelet granules and PMPs might promote tumor cell proliferation (96), noting that anti-proliferative effects of PMPs were also reported (219). These data raise questions of whether platelet pro-angiogenic and pro-mitogenic effects might impact tumor progression, highlighting the need of further research in this field.

In addition Magnus et al. have shown that TF expression by tumor cells is associated with exit from tumor cell dormancy (220), but its possible role in metastatic dormancy is not clear. Effects of TF on metastatic dormancy might be due to intracellular signaling downstream of the TF receptor but could be completely independent of tumor cell-platelet interactions. Nevertheless, Albrengues et al. have elegantly shown that NETs support the awakening of dormant disseminated tumor cells in the lungs by cleaving subendothelial laminin, which in turns interacts with α_3_β_1_ integrin on dormant cancer cells and induces cell cycle progression (221). Taking into consideration that activated platelets induce NETosis through soluble mediators (184), sustained and systemic platelet activation in cancer patients might contribute to cancer recurrence through activation of dormant cells. Further research is needed to determine whether there is a contribution of platelets to metastatic dormancy.

Altogether, platelets interacting with a tumor cell are active biosuppliers of many assets needed to survive in the blood stream, adhere to the endothelium and extravasate. Hence, cancer cells that can induce the deposition of microclots on their surface have the potential to initiate metastasis, irrespective of cancer type, site of metastasis, and oncogenic mutations. This might explain the widespread pro-metastatic effect of platelet activation across many different cancer types.

## Megakaryocytes and Metastasis

Whereas, the contribution of platelets to metastasis has been extensively characterized, the role of their precursor megakaryocytes is less well-defined. In general, cancer is associated with increased megakaryopoiesis. Mice with ovarian tumors have higher numbers of bone marrow and spleen megakaryocytes, which correlated with their increased platelet counts. A similar correlation between bone marrow megakaryocytes and platelet count is found in women with ovarian cancer (33). Higher counts of bone marrow megakaryocytes, pro-platelets and platelets were found in pediatric chronic myeloid leukemia (222) and in metastatic breast cancer (223). Higher numbers of pulmonary megakaryocytes were also observed in patients with lung metastases (224). The increase of megakaryocytes during neoplasia might be partially traced back to TPO, whose plasma concentration is significantly higher in cancer patients and is predictive of poorer response and survival (225, 226). Tumor cells might directly secrete TPO (227) or alternatively induce its production by stroma cells. In ovarian cancer, interleukin-6 (IL-6) produced by the primary tumor stimulates the production of TPO by hepatic cells, the physiological source of TPO, which in turn induces megakaryocyte maturation and platelet production in the bone marrow (33), as a systemic effect and possibly prior to metastatic dissemination. Similarly, highly metastatic mammary adenocarcinomas are associated with increased numbers of bone marrow megakaryocytes in rats, yet in the absence of bony metastases (228). Other pre-clinical evidence suggests that bone marrow megakaryopoiesis takes place after tumor cell colonization. Jackson et al. have shown that the number of megakaryocytes in the bone marrow increased after intracardial injection of breast cancer cells in mice (metastatic cancer to bones), but not after orthotopic injection in the mammary fat pad (localized cancer) (223). They further showed that tumor cell interaction with osteoblasts led to the release of soluble factors that induced proliferation of megakaryocytes (223).

Megakaryocytes are found in the main sites of blood-borne metastasis such as bone marrow, lungs and liver, as well as the circulation (229). Evidence is pointing to a role for megakaryocytes in cancer metastasis, although both pro- and anti-metastatic effects have been observed. Shirai et al. have shown that reduction of TPO synthesis in hepatic cells through silencing of the *THPO* gene results in slower metastatic progression of PyMT mammary tumors in mice and in lower numbers of metastatic lung nodules (230). In contrast, Tpo^−/−^ mice lacking more than 90% of bone marrow megakaryocytes develop more widespread and aggressive metastasis than their wild type counterpart (223), suggesting an anti-metastatic role of megakaryocytes. On the same line, TPO-driven megakaryopoiesis in the bone marrow decreases the incidence and size of tumor bone lesions (231). Interestingly, the number of circulating megakaryocytes has a trend to be associated with better survival and lower risk of metastatic disease in prostate cancer patients (229). There are different possible explanations for these contrasting roles of megakaryocytes during metastasis. On one hand, megakaryocytes can support metastasis through thrombopoiesis, and thrombocytosis is associated with cancer progression (16–20). Stone et al. have shown that paraneoplastic thrombocytosis in cancer patients can result from increased megakaryopoiesis through the IL-6/TPO axis (33). Importantly, high concentrations of plasma IL-6 (>10 pg/mL) are associated with lower overall patient survival, and inhibition of IL-6 synthesis by tumor cells restores normal platelet numbers and improves disease control (33). Similarly, an anti-IL-6 monoclonal antibody (sarilumab) used for treatment of rheumatoid arthritis is associated with a decreased platelet count (232). On the other hand, megakaryocytes can support the formation of (pre-)metastatic niches that affect tumor skeletal growth. In the bone marrow vascular niche, megakaryocyte crosstalk with osteoblasts, osteoclasts, and endothelial cells controls bone homeostasis [as reviewed by (37)]. Megakaryocytes release a plethora of osteoblast growth factors, such as fibroblast growth factor (FGF)-2 and TGF-β (37), that can support metastatic growth. Concomitantly, megakaryocytes suppress bone resorption by inhibiting osteoclast activity, with possible anti-metastatic consequences due to reduced release of bone matrix-bound growth factors and suppression of the well-described vicious cycle of osteolytic bone metastasis (233). Additionally, megakaryocytes store in their granules and secrete an array of pro- and anti-angiogenic factors (37, 213, 214, 216), which could control the vascularity of bone niches during skeletal metastasis. During bone metastasis, tumor cells hijack this crosstalk. In tumor bearing-mice, mature bone marrow megakaryocytes, and platelets express and release higher levels of the anti-angiogenic thrombospondin (TSP)-1 (234). Furthermore, the direct contact of megakaryocytes with prostate cancer cells induces cell cycle arrest and apoptosis (231). Hence, the direct interface of megakaryocytes with tumor cells can result in both pro- and anti-metastatic consequences.

In conclusion, the role of megakaryocytes during metastasis has only started to be appreciated. Although separating the direct effect of megakaryocytes on disseminated tumor cells from their thrombopoietic function will be challenging, further research is needed to reconcile the observed pro- and anti-metastatic effects of megakaryocytes and to understand the role of megakaryocytes on metastasis to secondary thrombopoiesis sites, such as lungs and liver.

## Hypercoagulability in Cancer Patients

A common complication of cancer is the onset of a hypercoagulable state that promotes thromboembolism (TE), which is the second leading cause of cancer-related morbidity and mortality (235, 236). Cancer patients have a 4- to 7-fold higher risk of TE than the general population (237), in particular if undergoing chemotherapy (238). Emboli in the circulation can lead to arterial thromboembolism (ATE), manifesting as myocardial infarction and ischemic stroke, and venous thromboembolism (VTE), which leads to events such as deep venous thrombosis and pulmonary embolism. Although ATE affects an average of 4.7% of all cancer patients (236), VTE events are far more common in cancer patients. The incidence of VTE varies between different types of cancer, with the highest incidence in pancreatic cancer (30–57% of patients) (239), followed by brain tumors (up to 31.7%) (240), and lung cancer (up to 21.5%) (241). Cancer-associated VTE causes a 3 to 10-fold higher risk of death and is consistently associated with worse prognosis, including lower overall survival and higher mortality (242). Advanced-stage tumors are largely associated with a greater risk of TE and cancer patients with TE have higher risk of tumor progression (236, 242). TE can be the first manifestation of an undiagnosed cancer and 90% patients with VTE have underlying metastatic cancer at the time of the event (108, 243). These associations highlight the link between thrombosis and the metastatic cascade and point to the pro-coagulant properties of metastatic cells as the underlying mechanism of VTE in cancer patients.

The expression of hemostatic factors and adhesion proteins in CTCs and tumor biopsies has been associated with poor prognosis. TF expression in malignant cells is an independent prognostic factor of tumor progression and VTE risk across a range of cancer types, including pancreatic (244, 245), glioma (246), colorectal cancer (247), breast cancer (248), and non-small cell lung cancer (249, 250). CD97 was highly expressed on CTCs from blood of metastatic prostate cancer and in their bone metastases (97). Similarly, expression of selectin ligands by tumor cells leads to a poorer prognosis (251).

Pro-thrombotic MPs have been proposed to play a major role in the pathogenesis of disseminated VTE in cancer patients. Not only do cancer patients have higher levels of circulating pro-coagulant MPs than healthy people, but cancer patients with VTE also have significantly higher plasma numbers of TF-expressing MPs and increased pro-coagulant MP activity in comparison to cancer patients without VTE (101, 108, 109, 252, 253). The concentration of TF-bearing MPs was associated with a higher risk of TE and higher mortality rate in some studies (101, 106, 108, 252), but not in others (110, 253). Also, the detection of TF^+^ MPs failed to predict the occurrence of TE events (252). Thus, the prognostic value of tumor-derived TF^+^ MPs remains controversial, suggesting the existence of TF-independent pathways of thrombosis induced by extracellular vesicles.

In contrast, a growing body of literature on PMPs supports their potential contribution to VTE in cancer patients. Previous reports have shown increased plasma levels of PMPs in cancer patients vs. healthy controls, with increased levels correlated with tumor grade (254–256). Importantly, high plasma PMP levels are correlated with poor overall survival from prostate cancer and higher risk of metastasis in both prostate and gastric cancer, where PMPs can be used as predictors of metastatic disease with high sensitivity and specificity (255, 257). Cancer patients with a history of VTE have higher plasma levels of PMPs than cancer patients with no prior VTE events (113, 256). These vesicles are derived from both resting and activated platelets and only a minority expressed TF (113, 256). Importantly, Bucciarelli et al. observed that PMPs could be an independent predictor of overall VTE risk, supporting the use of PMPs as a biomarker to identify patients at high risk of VTE, with or without cancer. Although further research is needed to understand the casual relationship between PMPs, VTE, and cancer metastasis, these reports suggest that plasma PMPs could be used as prognostic factors for cancer diagnosis, progression and VTE occurrence, and might be employed to guide the implementation of preventive thromboprophylaxis in high risk patients, irrespective of cancer status.

## Drug Platelets, Drug Metastasis?

Considering the central role of platelets in the onset of metastasis and VTE, it follows that anti-coagulant drugs might be used to prevent VTE or metastasis altogether. Several families of anti-coagulant drugs that can target different aspects of the coagulation cascade, including thrombosis (aspirin and antagonists of P2Y_12_ and PARs) and coagulation factors (heparins, factor X inhibitors), have been evaluated in clinical trials as possible adjuvant therapies for cancer patients (Table 1). By affecting the normal hemostatic process, these drugs are often associated with side effects such as hemorrhage (275, 276) and therapies with higher efficacy, safety, and ease of administration have been evaluated over time.

**Table 1:** List of clinical trial comparing the effect of adjuvant anti-coagulant drugs to control (no-treatment or placebo treatment) in terms of cancer progression, metastatic disease, and survival.

**Family of drugs and mechanism of action**	**Drug tested**	**Clinical design**	**Treatment arms**	**Effect on cancer progression**		**Effect on survival**		**Reference**
				**Observation**	**Outcomes**	**Observation**	**Outcomes**	
**Vitamin K antagonists** Inhibition of Vitamin K epoxide reductase, limiting activation of vitamin k-dependent coagulation factors.	Warfarin	Randomized control clinical trial. *n* = 431 patients with solid cancers.	Warfarin (*n* = 215); no treatment (*n* = 216).	No difference in disease-free survival and incidence of disease progression, with the exception of small cell lung cancer patients. More metastasis (12 sites) and less metastasis (14 sites) in the warfarin group vs. control group.		No difference in survival, with the exception of small cell lung cancer patients.	OS = 49.5 weeks (warfarin) vs. 23 weeks (control)	(258)
	Warfarin	Prospective randomized trial. *n* = 294 patients with small cell lung cancer receiving chemotherapy, followed up for 36 months.	Warfarin (*n* = 103); no anti-coagulants (*n* = 86).	Higher rate of disease free survival.	CR = 17% (warfarin) vs. 8% (control)	Prolonged survival.	OS = 9.3 months (warfarin) vs. 7.9 months (control)	(259)
	Warfarin	Randomized clinical trial. *n* = 311 women with metastatic breast cancer receiving chemotherapy.	Warfarin (*n* = 152); placebo (*n* = 159).			No difference in survival.	Survival rate = 57% (warfarin) vs. 63% (placebo)	(260)
	Warfarin	Randomized clinical trial. *n* = 183 patients with small cells lung cancer receiving chemotherapy, followed up for 69 months.	Warfarin (*n* = 91); no anti-coagulants (*n* = 92).	No difference in disease-free survival rate (CR) and duration (DFS). Non-significant increased rate of distant relapse.	CR = 74% (warfarin) vs. 83% (control) DFS = 13.7 months (warfarin) vs. 24.0 months (control) Rate of relapse = 33% (warfarin) vs. 19% (control)	Non-significant increase in survival.	OS = 21.4 months (warfarin) vs. 18.6 months (control)	(261)
	Warfarin or acenocoumarol	Observational cohort study. *n* = 76,008 patients, observed for 8.2 years.	VKAs (*n* = 3,231); controls (*n* = 72,777).	No effect on cancer progression.	RR = 0.85 (95% CI, 0.65–1.12)	Higher mortality.	*HR* = 1.12 (95% CI, 1.05–1.19)	(262)
	Warfarin	Meta-analysis of the Finnish Randomized Study of Screening for Prostate Cancer. *n* = 6, 537 men with prostate cancer, followed up for 14 years.	Warfarin (*n* = 1,210); no anti-coagulants (*n* = 5,327).	Higher risk of high-grade cancer. Higher risk of metastatic cancer.	*HR* = 1.11 (95% CI, 1.04–1.36) *HR* = 1.48 (95% CI, 1.01–2.17)			(263)
**Low molecular weight heparins** Activation of antithrombin III, leading to inhibition of factor Xa and thrombin.	Dalteparin	Randomized clinical trial. *n* = 84 patients with small cell lung cancer.	Dalteparin (*n* = 42); no anti-coagulants (*n* = 42).	Increased progression-free survival rate.	1–2 year PFS rate = 30.4–3.4% (dalteparin) vs. 11.7–0% (control)	Increased survival. Reduced risk of death.	1–2 year OS rate = 51.3–17.2% (deltaparin) vs. 29.5–0.0% (control) *HR* = 0.56 (95% CI, 0.3–0.86)	(264)
	Dalteparin	Randomized double-blind placebo-controlled trial. *n* = 385 patients with advanced cancer.	Dalteparin (*n* = 190); placebo (*n* = 184).			Increased survival.	1–2–3 year OS rate = 46–27–21% (deltaparin) vs. 41–18–12% (control)	(265)
	Nadroparin	Double-blind study. *n* = 302 patients with advanced solid tumors, followed for up to 1 year.	Nadroparin (*n* = 148); placebo (*n* = 154).			Reduced overall mortality.	*HR* = 0.75 (95% CI, 0.59–0.96)	(266)
	Nadroparin	Randomized open-label study. *n* = 503 patients with non-small-cell lung cancer (stage IIIB), hormone-refractory prostate cancer or locally advanced pancreatic cancer.	Nadroparin (*n* = 244); no anti-coagulants (*n* = 259).	No effect on cancer progression.	Time to progression = 5.0 months (nadroparin) vs. 5.8 months (control)	Non-significant prolonged survival. No difference in overall mortality.	Median survival = 13.1 months (nadroparin) vs. 11.9 months (control) *HR* = 0.86 (95% CI, 0.67–1.10)	(267)
	Nadroparin	*Post-hoc* analyses of randomized double-blind placebo-controlled trial. *n* = 1,166 ambulatory patients with solid cancer, followed up for 111–113 days.	Nadroparin (*n* = 779); placebo (*n* = 387).			Increased survival in patients with disease control.	1-year survival rate = 83% (nadroparin) vs. 76% (placebo)	(268)
	Deltaparin	Randomized clinical trial. *n* = 2,202 patients with lung cancer, followed up for 23.1 months.	Dalteparin (*n* = 1,101); no anti-coagulants (*n* = 1,101).	No difference in the risk of metastasis. No difference in metastasis-free survival.	*HR* = 0.99 (95% CI, 0.91–1.08) *HR* = 1.01 (95% CI, 0.93–1.1)			(269)
	Tinzaparin	Randomized clinical trial. *n* = 549 patients with non-small cell lung cancer, followed up for 5.7 years.	Tinzaparin (*n* = 269); no anti-coagulants (*n* = 280).			No difference in overall survival.	*HR* = 1.24 (0.92–1.68)	(270)
**NSAIDs** Cyclooxygenase inhibitors. COX-1 inhibition results in an anti-thrombotic effect, COX-2 inhibition results in an anti-inflammatory effect.	Aspirin	Meta-analysis of case-control studies. *n* = 141,577 patients with cancer exposed or not to aspirin.		Reduced risk of cancer with distant metastasis. No difference in risk of regional spread.	*OR* = 0.69 (95% CI, 0.57–0.83) *OR* = 0.98 (95% CI, 0.88–1.09)			(271)
	Aspirin	Meta-analysis of randomized controlled trials. *n* = 17,285 patients with or without cancer, treated with aspirin (>75 mg/day) or placebo.		Reduced risk of cancer with distant metastasis, irrespective of initial diagnosis. Reduced risk of metastasis in patients with no metastasis at initial diagnosis.	*HR* = 0.74 (95% CI, 0.48–0.84) *HR* = 0.45 (95% CI, 0.28–0.72)	Lower rate of death due to cancer.	*HR* = 0.71 (95% CI, 0.57–0.90)	(22)
	NSAIDs	Meta-analysis of previous clinical studies. *n* = up to 247,826 patients with cancer exposed or not exposed to NSAIDs.		Reduced risk of distant metastasis. Slightly reduced risk of lymph node metastasis.	RR = 0.623 (95% CI, 0.515–0.753) RR = 0.949 (95% CI, 0.914–0.985)			(272)
	Celecoxib	Randomized control trial. *n* = 200 patients with metastatic or advanced gastric cancer.	Celecoxib (*n* = 100); no anti-coagulants (*n* = 100).	Longer progression-free survival of COX-2+ patients.	PFS = 7.5 months (celecoxib) vs. 5 months (control)	Longer overall survival of COX-2+ patients.	*OS* = 14 months (celecoxib) vs. 10 months (control)	(273)
**Mixed anti-coagulants**	Anti-coagulant therapy (aspirin, warfarin, clopidogrel, and enoxaparin).	Case-control study. *n* = 5,955 patients with prostate cancer, followed up for 70 months.	Anti-coagulants (*n* = 2,175); no anti-coagulants (*n* = 3,780).	Lower risk of disease recurrence. Lower risk of bone metastasis.	7–10 year disease recurrence rate = 24–28% (anti-coagulant) vs. 28–36% (control) 7–10 year bone metastasis rate = 1–3% (anti-coagulant) vs. 3–6% (control)	Lower risk of prostate cancer specific mortality. Lower risk of prostate specific mortality in the aspirin-user group.	7–10 year mortality rate = 1–3% (anti-coagulant) vs. 3–8% (control) *HR* = 0.43 (95% CI, 0.21–0.87)	(274)

*Patients were treated with concomitant chemotherapy, radiotherapy, and/or surgery as described in the referred papers. Clinical trials with VTE as only endpoint or comparing different anti-coagulants are not listed here. CR, complete response; DFS, disease free survival; HR, hazard ratio; OR, odds ratio; RR, risk ratio; CI, confidence intervals*.

Historically, vitamin K antagonists (VKAs) such as warfarin were employed as the standard of care in cancer patients when they were the only anticoagulant pharmaceutical available. Despite promising pre-clinical data, long-term use of VKAs failed to show any effect on the risk of metastatic disease in most studies (258–263), and was generally associated with a higher risk of bleeding and higher mortality rate than other anticoagulants (277, 278). Low molecular weight heparins (LMWHs) were introduced later on. They showed a higher efficacy than unfractionated heparins and could be self-administered subcutaneously at home instead of intravenously in hospitalized patients (279). LMWHs were found to reduce cancer-related mortality and prolong survival of cancer patients with better prognosis at the start of randomization [e.g., disease control; (265, 266, 268)], consistent with its prevention of metastatic disease. However, increased overall survival and reduced VTE recurrence due to LMWHs has only been identified in some studies (264, 266, 267), and not others (269, 270), questioning further clinical evaluation of LMWHs as possible metastasis/VTE-preventive agents. These results could be partially due to the advanced cancer stage of enrolled patients, where pre-existing occult metastasis could not be affected by anti-coagulant therapy. Additionally, the anti-metastatic effect of LMWHs might be equally due to their inhibition of coagulation factors (e.g., thrombin and factor Xa) and their binding to selectins and integrins, affecting interactions between tumor cells, immune cells, and the vascular wall directly (280). The pleiotropic effects of LMWHs make it difficult to draw definitive conclusions from these trials.

More recently, other anticoagulants have also shown discouraging results in clinical trials. Despite promising pre-clinical data, the ADP P2Y_12_ receptor antagonist prasugrel (TRITON-TIMI 38 trial) (281, 282) and the P2Y_12_ inhibitors thienopyridines (clopidogrel and prasugrel) (283) increased the incidence of newly diagnosed solid cancers and the risk of cancer-related death. Similar effects were seen upon treatment with the PAR1 antagonist vorapaxar (TRACER trial) (284) and the factor Xa inhibitor apixaban (APPRAISE-2 trial) (285). The reason of this effect is still not clear, and additional data are needed to address the safety of these and other anticoagulant drugs.

Less controversial results come from clinical trials evaluating NSAIDs as an adjuvant therapy for cancer patients, which have been overall associated with longer progression-free survival and reduced risk of distant metastasis (271–274, 286). The strongest case is presented by the cyclooxygenase (COX)-1 and−2 inhibitor aspirin. Long-term aspirin treatment (≥5–10 years) has proven to reduce cancer incidence and mortality, in particular due to reduction in colorectal adenomas and cancer (22, 287–291). This is possibly related to the inhibitory effect of aspirin on COX-2, which is involved in the early carcinogenesis of colorectal adenocarcinoma (292), or to other COX-independent targets that are affected by higher doses of aspirin. Aspirin treatment is also associated with an increased T cell infiltration in ovarian cancer, potentially paving the way to use aspirin in combination with checkpoint inhibitors for tumor control (293). In addition to its effect on primary cancers, the meta-analysis of case-control studies and randomized control trials by Rothwell et al. has shown that regular use of aspirin reduced the risk of metastatic cancer, particularly pulmonary metastasis (271, 286). Results were particularly impressive for patients with no evidence of metastasis at randomization, for whom the risk of developing in-trial metastasis was more than halved (*HR* = 0.45, 95% CI 0.28–0.71, *p* = 0.0009). Further this reduction occurred in many cancer types for which COX-2 is not known to be important in its carcinogenesis. The risk of cancer-related deaths was significantly reduced by low-dose aspirin (<300 mg/day), which is mainly anti-thrombotic, but not high-dose aspirin (≥300 mg/day), which is both anti-thrombotic and anti-inflammatory (286), reinforcing the hypothesis that aspirin prevents metastasis through its effect on platelet aggregation. More recently, Yang et al. have found a reduced number of blood CTCs in colorectal cancer patients treated with aspirin, consistent with its effect on tumor cell survival in the circulation and metastatic seeding (294). Our group has conclusively shown that this metastasis-preventive effect of aspirin relies on the inhibition of COX-1 and its downstream product TXA_2_ in platelets, which is responsible for the generation of a favorable intravascular niche promoting the survival and metastatic seeding of disseminating tumor cells (9). Importantly, we showed that untreated mice infused with aspirin-treated platelets harbor significantly fewer lung metastasis, supporting the notion that the anti-metastatic effect of aspirin does not rely on extra-platelet targets. In light of these results, we propose that more selective inhibitors of TXA_2_, such as the dual TXAS inhibitor and TP antagonist picotamide, would achieve the same anti-metastatic result while sparing other gastroprotective COX-1 products, resulting in a better anti-thrombotic profile and less side effects. Future clinical trials might address the efficacy and safety of picotamide as adjuvant therapeutic intervention in cancer patients.

Other families of anti-platelet drugs, such as inhibitors of α_IIb_β_3_ (e.g., tirofiban and eptifibatide) and selectins (e.g., crizanlizumab and rivipansel), have FDA indications for the treatment of cardiovascular events, but their effect on cancer progression has not been evaluated yet. Additionally, reduction of paraneoplastic thrombopoiesis through IL-6 neutralization might represent a valuable approach to reduce metastatic spreading (33).

In conclusion, clinical trials so far have shown that there is no linear relationship between anticoagulation and metastasis prevention, but the effect largely depends on the drug used, the cancer type and the risk-benefit balance. These considerations have delayed the introduction of anticoagulant drugs as adjuvant therapy in cancer patients. A relatively weak and low-cost anticoagulant like aspirin has been by far the most successful drug in reducing the risk of metastatic cancer, in addition to reducing cancer incidence. It is possible that the inhibition of platelet COX-1/TXA_2_ signaling achieved by aspirin and TXA_2_ inhibitors, but not other anti-platelet drugs, is essential to disrupt the formation of intravascular pro-metastatic niches, which are required for early metastatic seeding. Although other anti-coagulant might affect later stages of metastasis, this effect might not be sufficient to achieve metastasis prevention (9). Although a more complete benefit-harm evaluation will have to be made in the future, the risk of major bleeding associated with long-term aspirin treatment, especially for exposure shorter than 5 years, might be counterbalanced by a reduced risk of vascular events and a significant reduction of cancer-related deaths (22). In 2015, the international Phase III clinical trial Add-Aspirin has started to address the effect of low (100 mg/day) or medium (300 mg/day) dose of aspirin or placebo on patients with non-metastatic solid tumors (breast, colorectal, gastro-esophageal, and prostate cancer) over a period of 5 years of more. Following up on excellent results on tolerability and toxicity, this trial will help understand the rate of survival, cancer recurrence, metastasis appearance, and the overall benefit-harm profile associated with aspirin use (295, 296).

## Conclusion and Future Perspectives

This review highlights the multifaceted interplay between tumor cells, megakaryocytes and platelets during hematogenous metastasis. Undoubtedly, the ability of tumor cells to interact with platelets confers a strong positive drive toward metastatic dissemination. The activation of platelets by tumor cells in the blood stream converts the physiologically “resting” intravascular niche into an active tumor-permissive environment, characterized by repressed immunosurveillance, and activated endothelial and myeloid cells. The advantage provided by platelets to the metastatic process can explain why cancers evolve to promote paraneoplastic megakaryopoiesis and thrombopoiesis to support their progression. The current knowledge on the contribution of platelets and megakaryocytes to metastasis opens promising therapeutic avenues. In particular, targeting platelets rather than the more genetically instable tumor cells might be a promising strategy for metastasis prevention. In support of this, experimental studies clearly indicate that abrogation of platelet interaction with tumor cells ultimately reduces metastasis across a wide range of cancer types and metastatic sites. We would expect thromboembolic complications to be reduced as well. How to target platelets, however, in cancer patient care is still a work-in-progress. For some anticoagulants, the extremely deleterious side effects due to the impairment of hemostasis is overshadowing any survival advantages associated with decreased cancer progression. Lessons from previous clinical trials suggest that the best strategy would be to target tumor cell-platelet interactions while leaving physiological platelet functions and stroma-platelet crosstalk unaffected. Further research is required to expand our understanding of the molecular mechanisms underlying these processes and to develop safer adjuvant or neoadjuvant therapies for cancer patients.

## Author Contributions

This manuscript was written by SL and RM. All authors contributed to the article and approved the submitted version.

## Conflict of Interest

The authors declare that the research was conducted in the absence of any commercial or financial relationships that could be construed as a potential conflict of interest.
